# Effect of amino acids and amines on the activity of the recombinant ι-carbonic anhydrase from the Gram-negative bacterium *Burkholderia territorii*

**DOI:** 10.1080/14756366.2021.1919891

**Published:** 2021-05-13

**Authors:** Viviana De Luca, Andrea Petreni, Vincenzo Carginale, Andrea Scaloni, Claudiu T. Supuran, Clemente Capasso

**Affiliations:** a Department of Neurofarba, Sezione di Scienze Farmaceutiche, Università degli Studi di Firenze, Polo Scientifico, Florence, Italy; b Proteomics & Mass Spectrometry Laboratory, Institute for the Animal Production System in the Mediterranean Environment, CNR, Naples, Italy; c Department of Biology, Agriculture and Food Sciences, CNR, Institute of Biosciences and Bioresources, Napoli, Italy

**Keywords:** Carbonic anhydrase, ι-class, activator, kinetics, amino acid, amine

## Abstract

We here report a study on the activation of the ι-class bacterial CA from *Burkholderia territorii* (BteCAι). This protein was recently characterised as a zinc-dependent enzyme that shows a significant catalytic activity (*k*_cat_ 3.0 × 10^5^ s^−1^) for the physiological reaction of CO_2_ hydration to bicarbonate and protons. Some amino acids and amines, among which some proteinogenic derivatives as well as histamine, dopamine and serotonin, showed efficient activating properties towards BteCAι, with activation constants in the range 3.9–13.3 µM. L-Phe, L-Asn, L-Glu, and some pyridyl-alkylamines, showed a weaker activating effect towards BteCAι, with *K_A_* values ranging between 18.4 µM and 45.6 µM. Nowadays, no information is available on active site architecture, metal ion coordination and catalytic mechanism of members of the ι-group of CAs, and this study represents another contribution towards a better understanding of this still uncharacterised class of enzymes.

## 1. Introduction

Enzyme activation implies that a chemical compound binding to an enzyme favourably affects the corresponding catalysed reaction rate^1^. Among the activators, worth mentioning are ions, small organic molecules (amines and amino acids, but also other derivatives), as well as peptides, proteins, and lipids^1^^,^^2^. Enzyme activation is classified as essential and non-essential. In the first case, the enzymatic reaction occurs only when the activator is present; in the second case, the catalysed reaction takes place with or without the activator^3^^,^^4^. Enzymatic reactions using ATP as substrate, such as those catalysed by kinases, are an excellent example of processes undergoing enzyme activation^5^. The suitable substrate for these biocatalysts is the complex formed by ATP and Mg^2+^ (the ion acting as activator), and the reaction does not take place when Mg^2+^ is absent and ATP is present^5^. In contrast, an elegant and well-described example of non-essential activation is represented by the superfamily of carbonic anhydrases (CAs, EC 4.2.1.1), which are widely investigated by us and others as drug targets^6–13^. These enzymes are involved in the catalysis of a pivotal physiological reaction, the reversible hydration of carbon dioxide to bicarbonate and protons^10^^,^^13–18^. Members of the CA superfamily are grouped into eight classes (α, β, γ, δ, ζ, η, θ and ι) according to their structural characteristics, and are distributed in all living organisms, starting from microorganisms to multicellular plants/animals^13–17^. For example, mammalian genomes encode only for numerous isoforms of the α-CA class and accomplish specialised functions in various tissues and organs^19–23^. In plants, α- and β-CAs have an essential role in photosynthesis and biosynthetic reactions related to it^9^. In simpler organisms, such as bacteria, Archaea and cyanobacteria, α-, β-, γ- and ι-CAs are present, which have a role in balancing the [CO_2_]/[HCO_3_^-^] ratio and the carbon dioxide fixation^9–11^^,^^13^^,^^18^^,^^24^. Marine diatoms encode for α-, δ-, ζ-, θ- and ι-CAs, which are involved in carbon dioxide fixation and metabolism^25–27^. In addition to α- and β-forms, protozoan species also expressed η-CAs. These enzymes are involved in *de novo* purine/pyrimidine biosynthetic pathways^28^. Finally, organisms of the fungal kingdom generally present enzymes of the β-class, which are present at least in one isoform^29–31^. Fungal CO_2_-sensing is directly stimulated by HCO_3_^−^, which is produced in a CA-dependent manner^31–34^.

The most extensively investigated CA activators (CAAs) belong to the compound groups of amines and amino acids^2^. The X-ray crystal structure of the human isoforms (hCA I and II) bound to activators, such as histamine, L-/D-histidine, L-/D-phenylalanine, D-tryptophan and others, allowed the comprehension of the activation mechanism and the structure-activity relationship governing it^2^^,^^35–41^. Contrary to most CA inhibitors (CAIs), such as anions and sulphonamides,^6^^,^^9^^,^^10^^,^^42^^,^^43^ CAAs bind to molecular regions at the entrance of the enzyme active site enhancing the proton transfer processes between the Zn^2+^-bound water molecule and the reaction medium; this is accomplished by a supplementary pathway provided by the proton-shuttling moieties of the activator^2^^,^^44^. As a result, CAAs increase the rate of the enzyme-catalysed process speeding up the proton transfer, which is the rate-determining step of the whole reaction, thus enhancing the catalytic efficacy of these enzymes (*k*_cat_ up to 10^6^ s^−1^)^2^^,^^44^. In the literature, the modulation of CAs activity through activators is less described than that by inhibitors; the latter is well documented for its relevance in the pharmacological field^20^^,^^42^^,^^45–48^. Nevertheless, CAAs, such as D-phenylalanine and imidazole, have been recently proposed as neuroenhancement drugs possibly improving synaptic efficacy, spatial learning and memory^2^^,^^49^. Moreover, it has been demonstrated that CA levels are significantly decreased in the brain of patients with Alzheimer’s disease (AD)^8^^,^^50^. These aspects corroborate the notion that CA modulators may have important applications in conditions in which individual learning and memory are impaired, such as aging or AD^8^^,^^39^^,^^50^^,^^51^. On the other hand, several CAIs (coumarins and their isosteres) were proved inhibiting CAs by occluding the entrance of the active site cavity; interestingly, their binding sites coincided with those observed for various CAAs^52^. In this regard, it is evident that investigations on CAAs and studies on the structure-activity relationship governing their action could have the advantage to improve the general design of novel CAs modulators, which can mimic the activator binding mode but can have an opposite effect on the enzyme activity, as seen for coumarins^52^. This is an important issue also in another pharmacological context, since it has been shown that the interference with bacterial CA activity can impair the microorganism growth and virulence, making the CA inhibition an exciting approach to contrast the emergence of antibiotic resistance associated with many infections^53^.

In analogy with what already done with other CAs classes from mammals and fungi^2^^,^^4^^,^^39^^,^^44^^,^^49^^,^^51^^,^^54^^,^^55^, we carried out an extensive study of the activation properties of amines and amino acids towards a member of the recently discovered group of *ι*-CAs. To this purpose, we investigated the effect of these CAAs on an enzyme identified in the genome of the non-pathogenic Gram-negative bacterium *Burkholderia territorii* recovered from groundwater samples (acronym BteCAι)^56^. This study is the first characterisation of the activation of a CA belonging to the *ι*-class.

## 2. Materials and methods

### 2.1. Reagents

Amines and amino acid derivatives **1–24** were obtained from Sigma-Aldrich (Milan, Italy) at the highest purity commercially available.

### 2.2. Cloning, production and purification of BteCAι

The protocol already developed and described by us^57^, involving enzyme cloning and expression in *Escherichia coli*, was here used to obtain a pure preparation of recombinant BteCAι. Briefly, The synthetic *B. territorii* gene encoding for the BteCAι was cloned into the expression vector pET100D-Topo/BteCAι and used to transform the Competent Escherichia coli BL21 (DE3) codon plus cells (Agilent). The cellular culture was induced with Isopropyl *β*-D-1-thiogalactopyranoside (IPTG) to overexpress the recombinant BteCAι. After the growth, the cells were harvested and disrupted by sonication. Cellular extract was purified using a nickel affinity column (His-Trap FF).

### 2.3. CA activation measurements

A Sx.18Mv-R Applied Photophysics (Oxford, UK) stopped-flow instrument was used to assay the CA-catalysed CO_2_ hydration activity^58^. Phenol red (at a concentration of 0.2 mM) was used as an indicator, working at the absorbance maximum of 557 nm, with 10 mM HEPES, pH 7.5 (for α-CAs and BteCAι)^59^^,^^60^ or 10 mM TRIS, pH 8.3 (for β-CAs)^61–64^ as buffers, containing 0.1 M NaClO_4_ (for maintaining constant ionic strength), following the CA-catalysed CO_2_ hydration reaction for a period of 10–100 s at 25 °C. The CO_2_ concentrations ranged from 1.7 to 17 mM to determine the kinetic parameters and activation constants. For each activator, at least six traces of the initial 5–10% of the reaction were used to determine the initial velocity. The uncatalyzed rates were determined in the same manner and subtracted from the total observed rates. Stock solutions of activators (at 0.1 mM concentration) were prepared in distilled-deionized water, and dilutions up to 1 nM were made thereafter with the assay buffer. Enzymes and activators were pre-incubated together for 15 min before the assay to allow the formation of the corresponding enzyme–activator complexes. The activation constant (*K_A_*) values, defined similarly to the inhibition constant counterparts, were obtained by considering the classical Michaelis–Menten equation (Equation (1), which was fitted by the non-linear least squares method using PRISM 3, and represent the mean from at least three different determinations:

(1) $$v=v_{\text{max}}/\left\{1+\left(K_{M}/\left[S\right]\right)\left(1+{\left[A\right]}_{f}/K_{A}\right)\right\}$$

where [*A*]*_f_* is the free concentration of the activator.

Working at substrate concentrations considerably lower than *K_M_* ([S] ≪*K_M_*), and considering that [A]_f_ can be represented in the form of the total concentration of the enzyme ([*E*]*_t_*) and activator ([*A*]*_t_*), the obtained competitive steady-state equation for determining the activation constant value is given by Equation (2):

(2) $$v=v_{0}.K_{A}/\left\{K_{A}+{\left({\left[A\right]}_{t}-0.5\{\left({\left[A\right]}_{t}+{\left[E\right]}_{t}+K_{A}\right)-{\left({\left[A\right]}_{t}+{\left[E\right]}_{t}+K_{A}\right)}^{2}-4{\left[A\right]}_{t}.{\left[E\right]}_{t}\right)}^{1/2}\right\}$$

where *v_0_* represents the initial velocity of the enzyme-catalysed reaction in the absence of activator^61–64^. This type of approach to measuring enzyme-ligand interactions is in excellent agreement with recent results from native mass spectrometry measurements^65^.

## 3. Results and discussion

### 3.1. Validation of BteCAι activity

**Figure 1.**
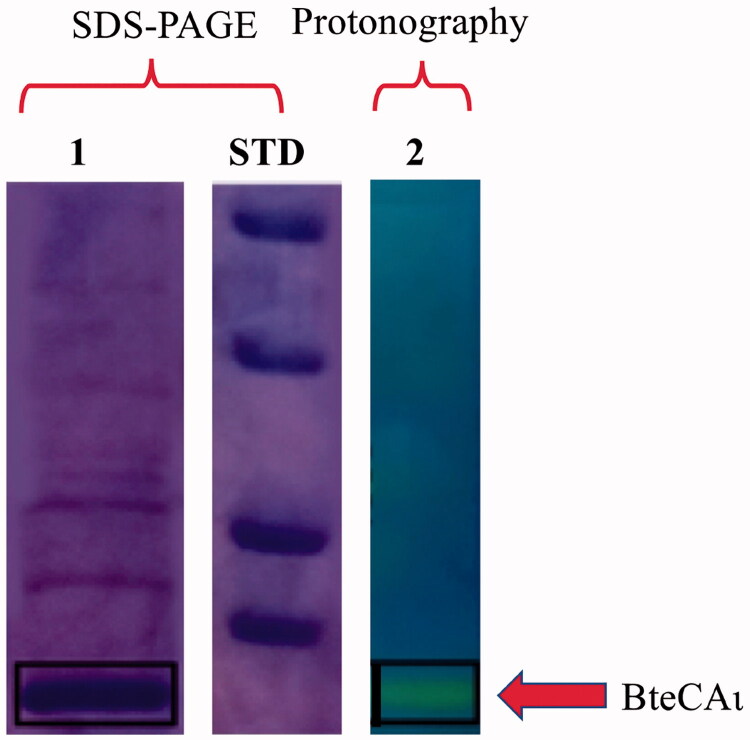
Combined lanes of SDS-PAGE and protonography of BteCAι. Lane 1, purified recombinant BteCAι; Lane 2, protonogram showing the enzyme activity on the polyacrylamide gel; Lane STD, molecular markers, from the top: 50.0 kDa, 37.0 kDa, 25 kDa and 20 kDa. Boxes with continuous lines indicate the protein bands identifying BteCAι (calculated molecular mass of 19.0 kDa).

BteCAι was heterologously expressed in *E. coli* and purified as already reported^57^. Enzyme preparation homogeneity, purity and *in gel* hydratase activity were verified using sodium dodecyl-sulfate-polyacrylamide gel electrophoresis (SDS-PAGE) and protonography, respectively (Figure 1).

The CO_2_ hydratase activity and the kinetic constants of purified, recombinant BteCAι were determined using the stopped-flow technique. Comparative experiments with human CA isoform I (hCA I) and isoform II (hCA II) were perfomed to relate results to well-known enzyme species. BteCAι showed a high catalytic activity (*k*_cat_ 3.0 × 10^5^ s *^−^* ^1^) for the physiological reaction of CO_2_ hydration to bicarbonate and protons and, as expected, was inhibited by the sulphonamide acetazolamide (*K_I_* = 519 nM) (Table 1).

**Table 1.** BteCAι, hCAI and hCAII kinetic parameters for the catalysed CO_2_ hydration reaction.

Organism	Enzyme acronym	Class	*k*_cat_ (s^−1^)	*K_M_* (M)	*k*_cat_/*K_M_* (M^−1^·s^−1^)	*K_I_* (Acetazolamide) (nM)
*Homo sapiens*	hCA I	α	2.0 × 10^5^	4.0 × 10^−3^	5.0 × 10^7^	250
	hCA II	α	1.4 × 10^6^	9.3 × 10^−3^	1.5 × 10^8^	12
*Burkholderia territorii*	BteCAι	*ι*	3.0 × 10^5^	3.1 × 10^−3^	9.7 × 10^7^	519

The kinetic measurements were carried out in 10 mM HEPES buffer, pH 7.5, at 20 °C.

Reported mean values are from three different assays performed by the stopped flow technique; errors were in the range of ±5–10% of the reported values (data not shown).

Worth mentioning is the fact that purified, recombinant BteCAι displayed a *k*_cat_ value of the same order of magnitude of hCA I, whereas the affinity for the substrate resulted higher than that of the two human isoforms (Table 1). Here, we underline that *ι*-CAs appear phylogenetically well separated from all the other bacterial CAs (α, β and γ), reinforcing the fact that these proteins were classified in a new CA class^66^. It has been speculated that bacterial ι-CAs may derive from the modification of an ancestor gene, which they had in common with γ-CAs; the latter enzymes are so far considered the oldest class among all CAs^9^^,^^10^^,^^15^^,^^18^^,^^66^. However, the hypothesis an ancestor gene common to ι- and γ-CAs needs to be validated by further work.

### 3.2. BtecAι activation profile

**Figure 2.**
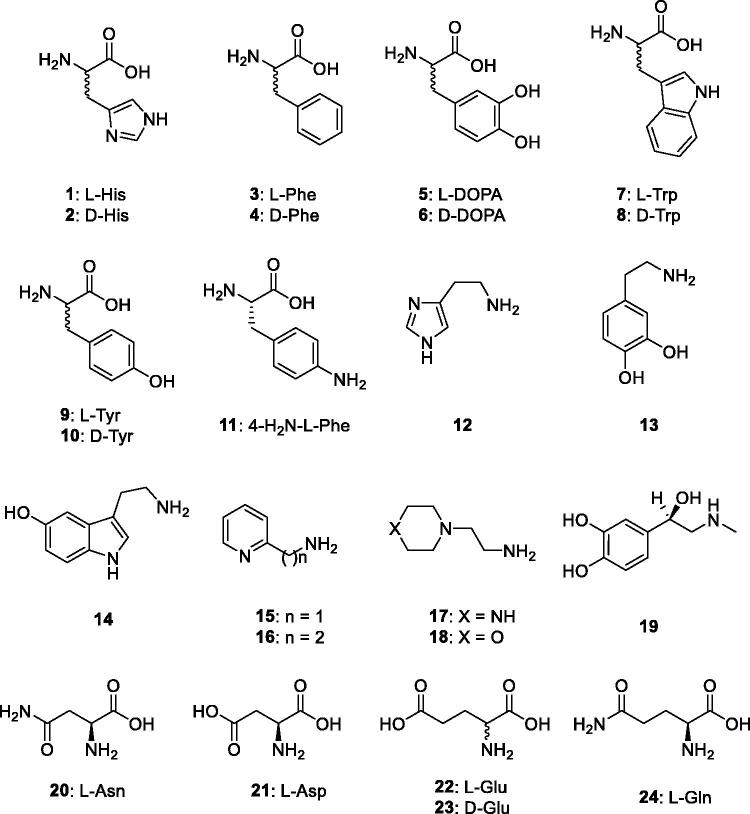
Amino acids and amines **1–24** investigated as CAAs of BteCAι.

Purified, recombinant BteCAι was then used to determine the corresponding activation profile with amines and amino acids (compounds **1–24**) reported in Figure 2. Some of these compounds are biogenic amines or bioactive derivatives that have a well-known pharmacological activity^67^. Resulting data provided original information on the activation profile of these CAAs with respect to a ι-CA class enzyme.

Table 2 shows kinetic parameters of some CAs in the presence of the substrate, namely CO_2_ at a concentration of 15 mM, when experiments were performed in the absence or presence of the activator L-Trp (at 10 µM final concentration). As expected, under these experimental conditions L-Trp efficiently activated hCA I, hCA II and β-CA from *Escherichia coli* (EcoCAβ); these enzymes were here used as positive controls since corresponding kinetic data and their structures in complex with CAAs have already been described^2^^,^^68^. Experiments originally demonstrated here that L-Trp can also exert an activation activity for a member of the ι-CA class. Indeed, the activator increased the *k*_cat_ value of all these enzymes but did not influence the corresponding *K_M_* one (data not shown). Also in the case of ι-CAs, this proved that, when the enzyme/L-Trp complex is formed, the latter takes part in the proton transfer process, but the activator does not interfere with the binding of CO_2_ to the protein active site, since the value of *K_M_* remained unchanged notwithstanding the absence/presence of L-Trp (Table 2).

**Table 2.** Activation of BteCAι, hCA I, hCA II and EcoCAβ with L-Trp. Experiments were performed for the CO_2_ hydration reaction, at 25 °C, using a stopped-flow assay^58^.

Enzyme acronym	Class	*k*_cat_*(s^−1^)	K_M_*(mM)	(*k*_cat_)_L-Trp_ (s^−1^)**	(*K_A_*)_L-Trp_ (µM)***
hCA I^a^	α	2.0 × 10^5^	4.0	3.4 × 10^5^	44.0
hCA II^a^	α	1.4 × 10^6^	9.3	4.9 × 10^6^	27.0
EcoCAβ^b^	β	5.3 × 10^5^	12.9	1.8 × 10^6^	18.3
BteCAι^c^	ι	3.0 × 10^5^	3.1	5.9 × 10^5^	10.2

*Observed catalytic rate without activator. *K_M_* values in the presence and the absence of activators were the same for the various CAs (data not shown).

**Observed catalytic rate in the presence of 10 µM L-Trp.

***The activation constant (*K_A_*) for each enzyme was obtained by fitting the observed catalytic enhancements as a function of the activator concentration^2^.

^a^Data for human recombinant isozymes, from ref. ^66^; ^b^data for bacterial recombinant enzyme, from ref. ^68^; ^c^This work.

All reported values are the mean from at least three determinations performed for the CO_2_ hydratation reaction; errors were in the range of ±5–10% of the reported values (data not shown)^58^.

Compounds **1–24** were thereafter assayed dose-dependently for their interaction with BteCAι with the aim to assess the corresponding activation constant (*K_A_*) values (Table 3). Again, the activation data of hCA I and II were analysed and are here reported for comparison reasons. The following structure-activity relationship (SAR) data for the activation of BteCAι may be noted from the data reported in Table 3:

**Table 3.** Activation of BteCAι, hCA I and hCA II with amino acids and amines **1–24**. Experiments were performed for the CO_2_ hydration reaction, at 25 °C, and performed by a stopped-flow assay^58^.

No.	Compound	*K_A_* (µM)*		
		hCA I^a^	hCA II^a^	BteCAι^b^
**1**	L-His	0.03	10.9	8.6
**2**	D-His	0.09	43	6.2
**3**	L-Phe	0.07	0.013	36.5
**4**	D-Phe	86	0.035	9.4
**5**	L-DOPA	3.1	11.4	4.3
**6**	D-DOPA	4.9	7.8	11.7
**7**	L-Trp	44	27	10.2
**8**	D-Trp	41	12	6.1
**9**	L-Tyr	0.02	0.011	8.0
**10**	D-Tyr	0.04	0.013	7.3
**11**	4-H_2_N-L-Phe	0.24	0.15	6.9
**12**	Histamine	2.1	125	6.0
**13**	Dopamine	13.5	9.2	8.7
**14**	Serotonin	45	50	13.3
**15**	2-Pyridyl-methylamine	26	34	24.1
**16**	2-(2-Aminoethyl)pyridine	13	15	21.5
**17**	1-(2-Aminoethyl)-piperazine	7.4	2.3	3.9
**18**	4-(2-Aminoethyl)-morpholine	0.14	0.19	12.0
**19**	L-Adrenaline	0.09	96.0	9.7
**20**	L-Asn	11.3	>100	45.6
**21**	L-Asp	5.20	>100	8.4
**22**	L-Glu	6.43	>100	18.4
**23**	D-Glu	10.7	>100	8.5
**24**	L-Gln	>100	>50	5.8

*Mean values from three determinations^58^. Errors were in the range of 5–10% of the reported values (data not shown).

^a^ Data for human recombinant isozymes, from ref.^2^.

^b^ This work.

- Most of the tested activators showed an efficient and rather flat activating efficacy towards BteCAι, with *K_A_* values ranging between 3.9 and 13.3 µM. Both amino acids (**1, 2, 4–11, 21, 23** and **24**) as well as amines (**12–14, 17–19**) showed this type of behaviour, with basically poor SAR to be discussed due to the low range of activity variations. This condition is different from what observed for hCA I and hCA II activation, for which some activators also showed nanomolar activity, e.g., L-Phe, L- and D-Tyr (hCA I and hCA II), and L-adrenaline, L- and D-His (hCA I). For the enantiomeric pairs L-/D-His, L-/D-Phe, L-/D-Trp and L-/D-Tyr, the D-enantiomer was always a better activator than the corresponding L-one. Only for DOPA the reverse was true, with L-DOPA being almost 3 times a more efficient activator compared to the corresponding D-enantiomer.
- Several compounds, among which L-Phe, the pyridyl-alkylamines **15** and **16**, as well as L-Asn and L-Glu, showed rather weak CA activating effects against the ι-class enzyme, with *K_A_* values ranging between 18.4 and 45.6 µM. Also for the L-/D-Glu enantiomeric pair, the D-enantiomer was always a better activator than the corresponding L-one. When data of the L-Asp/L-Asn pair were evaluated, the amide derivative appeared a 5.42-fold weaker activator than the acid counterpart. These results highlight that rather small structural changes (even at the stereogenic center) in the assayed compounds can lead to rather important modifications of the corresponding CA activating properties.
- As already anticipated above, the activation profile of the ι-class bacterial enzyme was very different from those of hCA I and hCA II.

## 4. Conclusions

In this study, we have reported an original analysis of the activation properties of various compounds towards the recently discovered group of *ι*-CAs. To this aim, we investigated the activation effect of amino acids and amines on BteCAι, which our group recently characterised as a zinc-dependent enzyme with a significant catalytic activity (*k*_cat_ 3.0 × 10^5^ s*^−^*^1^) for the physiological reaction of CO_2_ hydration to bicarbonate and protons. Some amino acids and amines, among which L-/D-His, D-Phe, L-/D-DOPA, L-/D-Trp, L-/D-Tyr, L-Asp, D-Glu, L-Gln, histamine, dopamine, serotonin, 1–(2-aminoethyl)-piperazine and others, showed efficient BteCAι activating properties, with activation constants ranging between 3.9 and 13.3 µM. Conversely, L-Phe, some pyridyl-alkylamines, L-Asn and L-Glu showed a weaker activating effect against this enzyme, with K_A_ values ranging between 18.4 and 45.6 µM. Although no information is available on active site architecture, metal ion coordination and catalytic mechanism of members of the ι-group of CAs yet, the results of this study can add further information for a better understanding of this novel class of enzymes. Their rationalisation will be fully achieved when the X-ray crystal structure of BteCAι and of a BteCAι-CAA complex will be solved, and mechanistic considerations will be then finally elaborated according to a structural basis.
